# Supplementary material for: Safety and Anatomical Accuracy of Dry Needling of the Quadratus Femoris Muscle: A Cadaveric Study
Source: Healthcare (Basel). 2025 Jul 26;13(15):1828. doi: 10.3390/healthcare13151828 (PMC12346365; doi:10.3390/healthcare13151828)
Supplement: Supplementary file 1 [file healthcare-13-01828-s001.zip › S1_DDN protocol.pptx]

## Slide 1
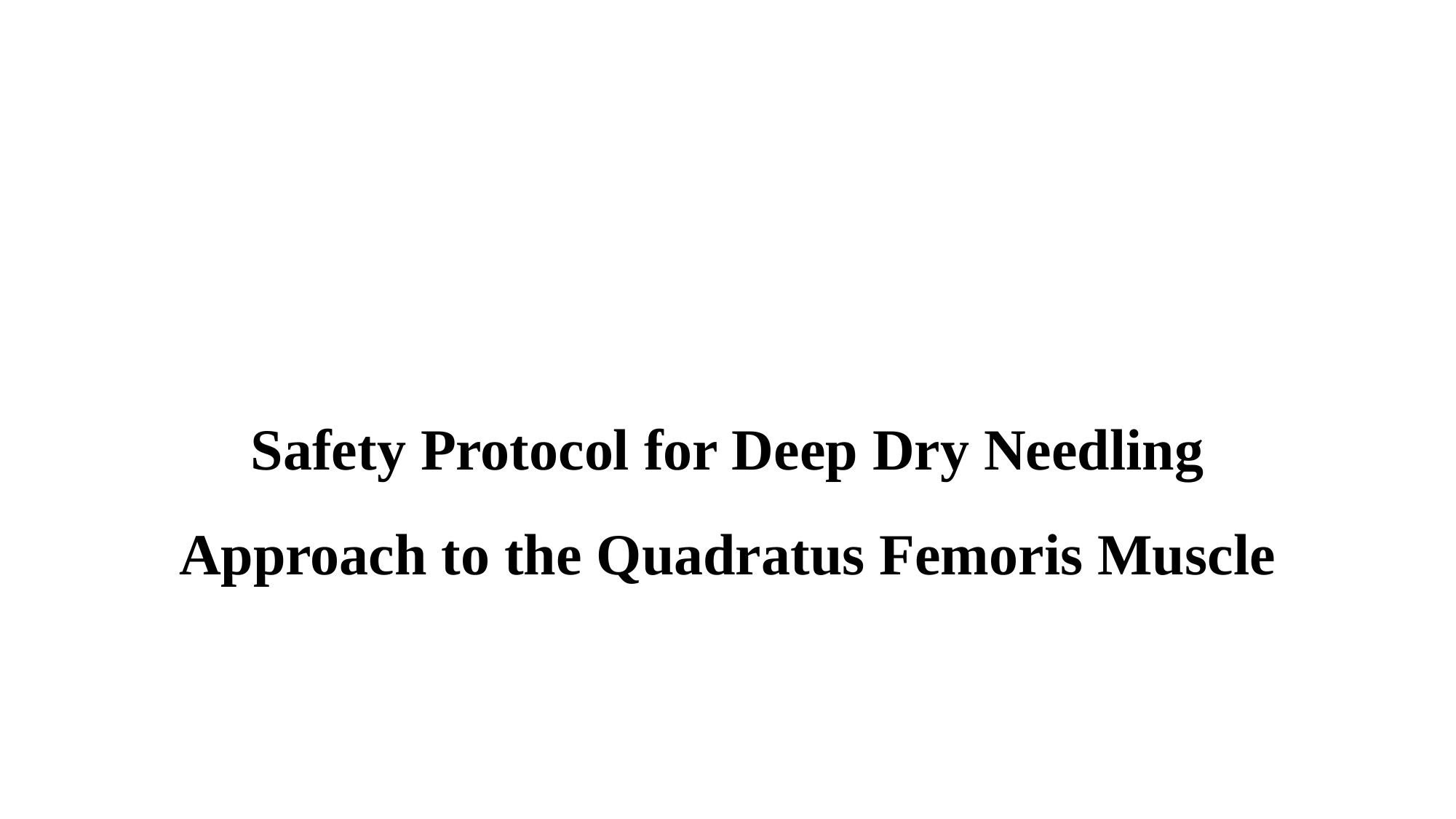

# Safety Protocol for Deep Dry Needling Approach to the Quadratus Femoris Muscle

## Slide 2
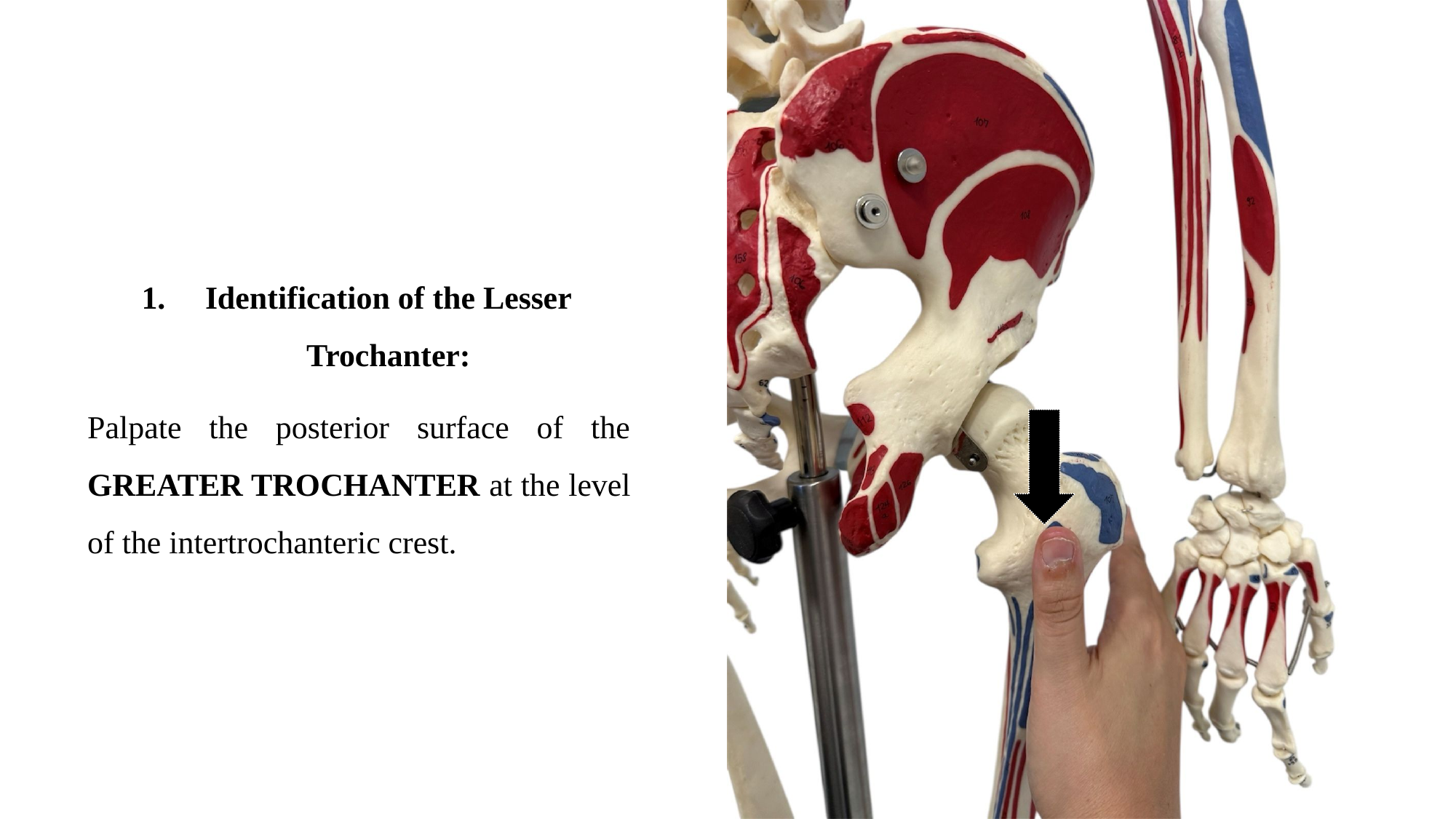

Identification of the Lesser Trochanter:
Palpate the posterior surface of the GREATER TROCHANTER at the level of the intertrochanteric crest.

## Slide 3
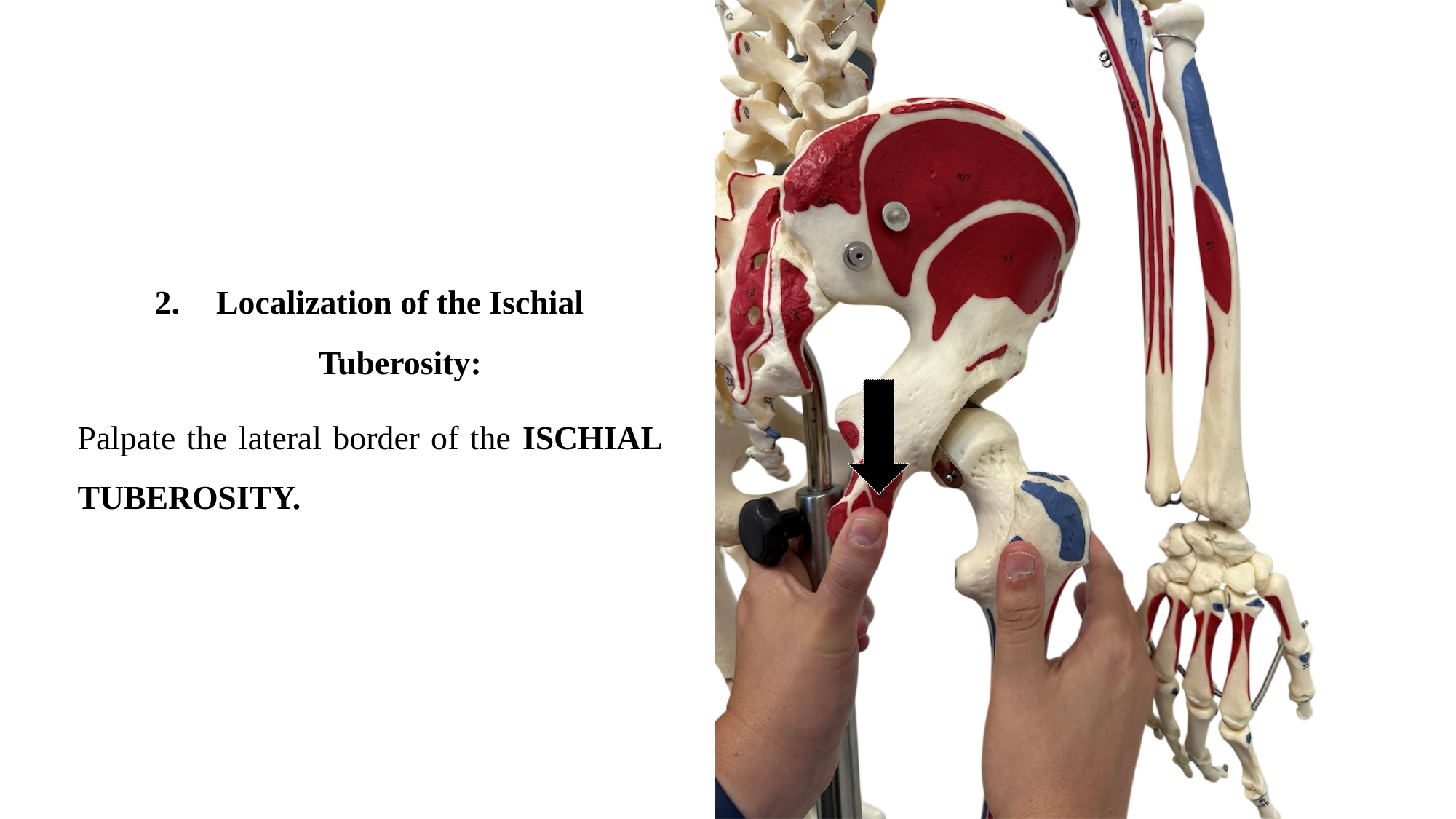

Localization of the Ischial Tuberosity:
Palpate the lateral border of the ISCHIAL TUBEROSITY.

## Slide 4
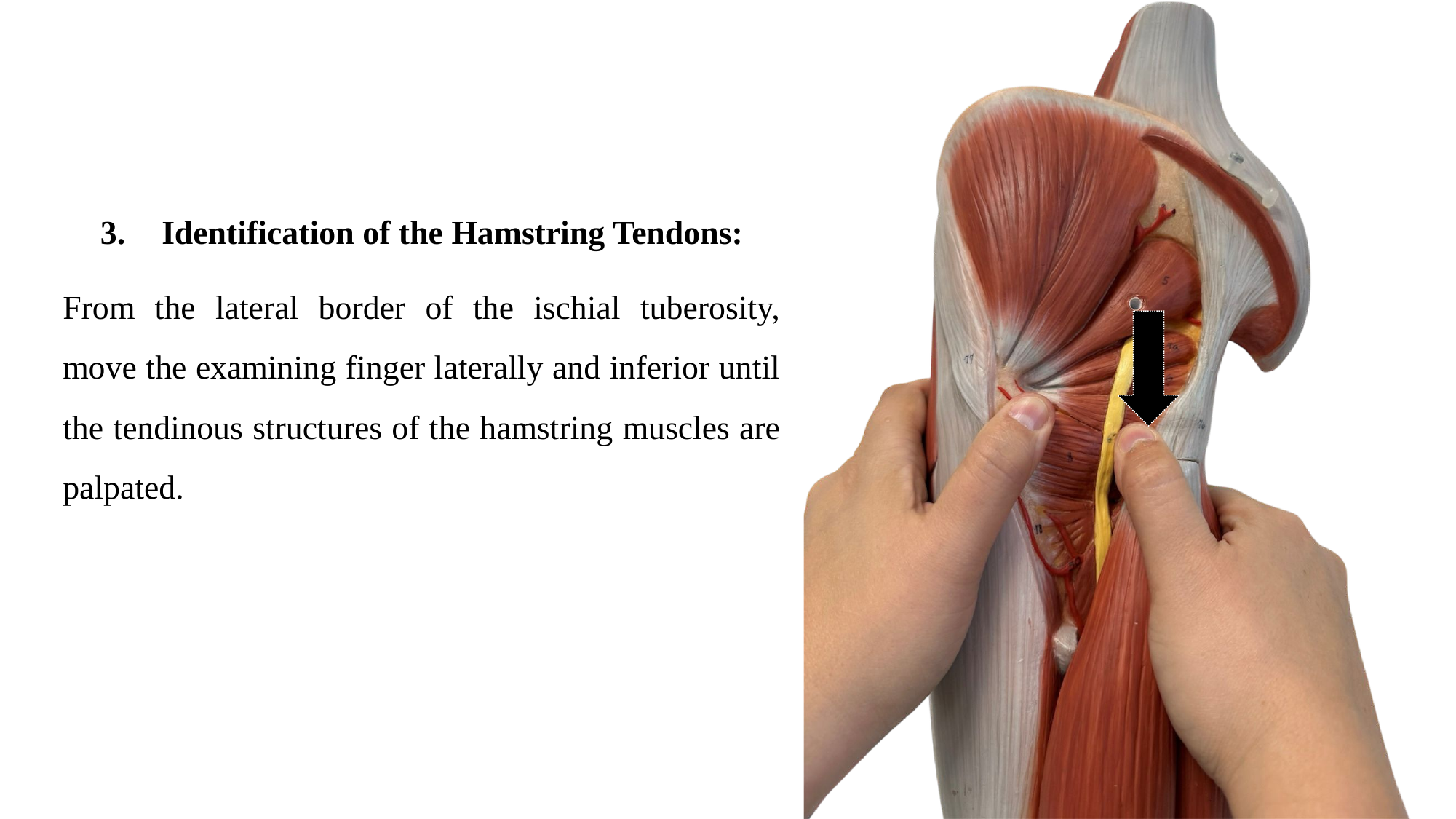

Identification of the Hamstring Tendons:
From the lateral border of the ischial tuberosity, move the examining finger laterally and inferior until the tendinous structures of the hamstring muscles are palpated.

## Slide 5
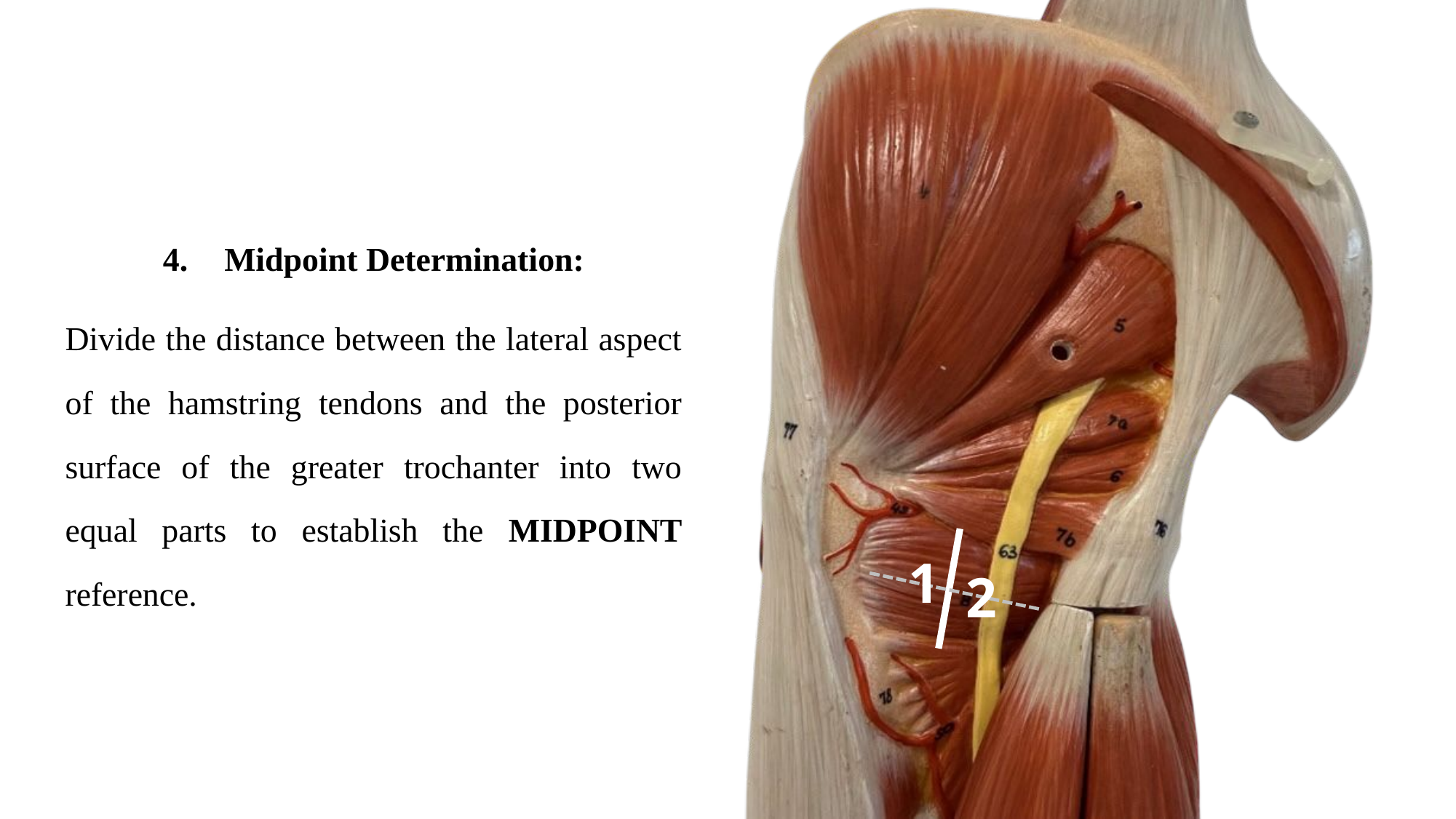

1
2
Midpoint Determination:
Divide the distance between the lateral aspect of the hamstring tendons and the posterior surface of the greater trochanter into two equal parts to establish the MIDPOINT reference.

## Slide 6
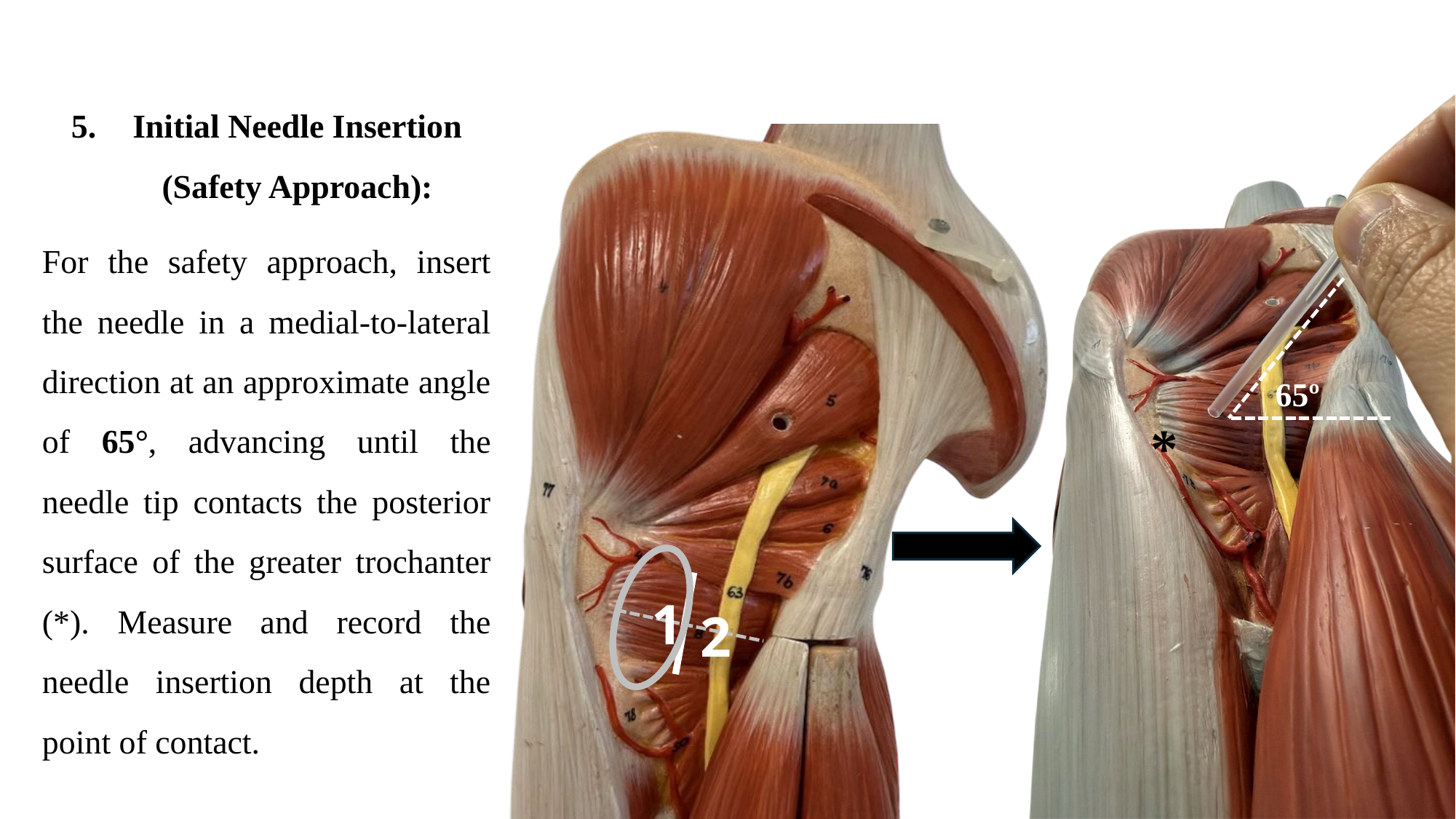

Initial Needle Insertion (Safety Approach):
For the safety approach, insert the needle in a medial-to-lateral direction at an approximate angle of 65°, advancing until the needle tip contacts the posterior surface of the greater trochanter (*). Measure and record the needle insertion depth at the point of contact.
1
2
65º
*

## Slide 7
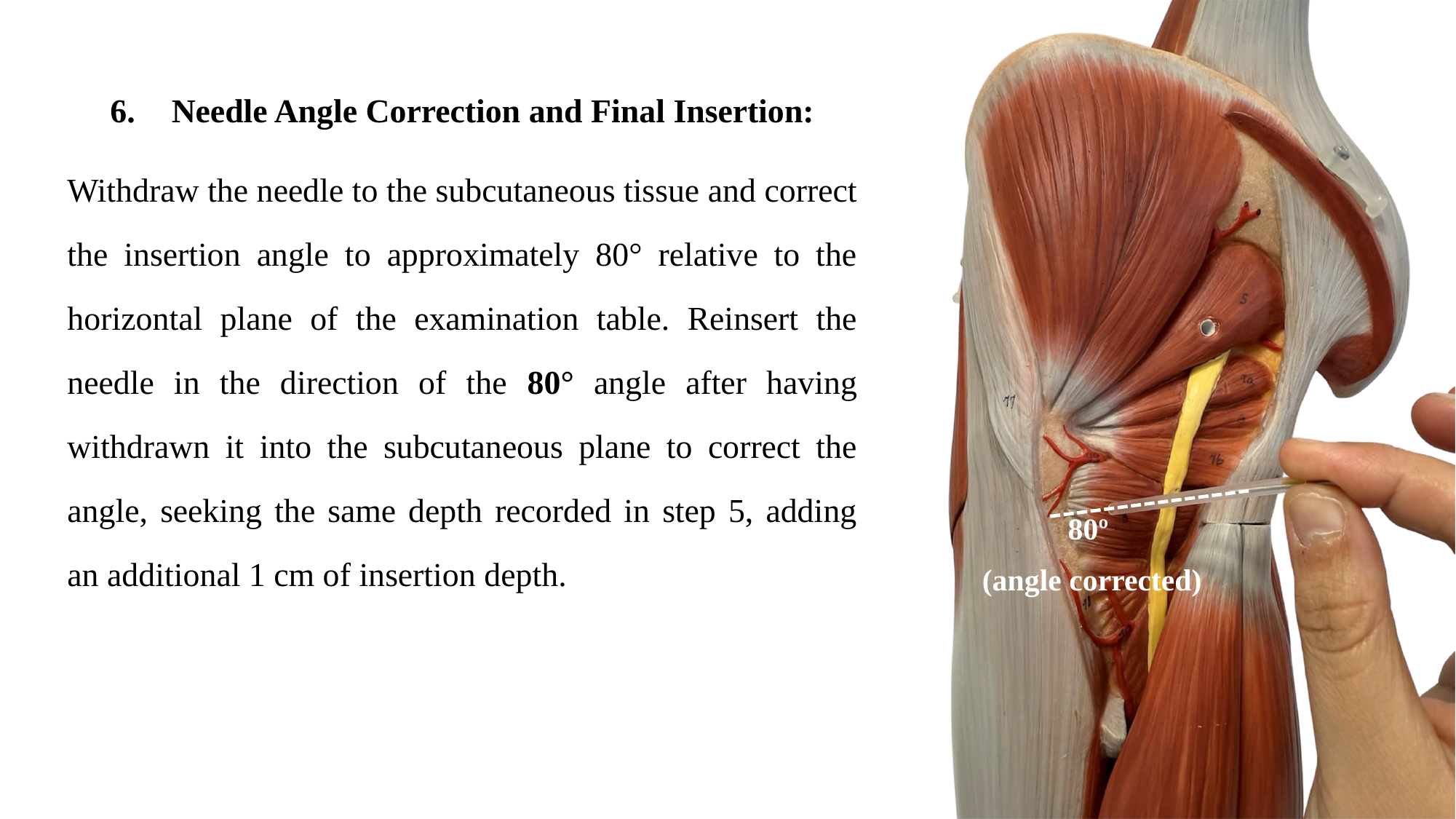

80º
(angle corrected)
Needle Angle Correction and Final Insertion:
Withdraw the needle to the subcutaneous tissue and correct the insertion angle to approximately 80° relative to the horizontal plane of the examination table. Reinsert the needle in the direction of the 80° angle after having withdrawn it into the subcutaneous plane to correct the angle, seeking the same depth recorded in step 5, adding an additional 1 cm of insertion depth.
